# Recent Advancements in Fabrication, Separation, and Purification of Hierarchically Porous Polymer Membranes and Their Applications in Next-Generation Electrochemical Energy Storage Devices

**DOI:** 10.3390/polym16233269

**Published:** 2024-11-24

**Authors:** Xiong Cheng, Joonho Bae

**Affiliations:** Department of Physics, Gachon University, Seongnam-si 13120, Gyeonggi-do, Republic of Korea

**Keywords:** hierarchically, porous membranes, electrochemical energy storage devices

## Abstract

In recent years, hierarchically porous polymer membranes (HPPMs) have emerged as promising materials for a wide range of applications, including filtration, separation, and energy storage. These membranes are distinguished by their multiscale porous structures, comprising macro-, meso-, and micropores. The multiscale structure enables optimizing the fluid dynamics and maximizing the surface areas, thereby improving the membrane performance. Advances in fabrication techniques such as electrospinning, phase separation, and templating have contributed to achieving precise control over pore size and distribution, enabling the creation of membranes with properties tailored to specific uses. In filtration systems, these membranes offer high selectivity and permeability, making them highly effective for the removal of contaminants in environmental and industrial processes. In electrochemical energy storage systems, the porous membrane architecture enhances ion transport and charge storage capabilities, leading to improved performance in batteries and supercapacitors. This review highlights the recent advances in the preparation methods for hierarchically porous structures and their progress in electrochemical energy storage applications. It offers valuable insights and references for future research in this field.

## 1. Introduction

The recent progress in hierarchically porous polymer membranes [1,2,3] has been both dynamic and promising, opening new avenues for their application in various fields. These membranes are characterized by their intricate, multilevel porous structures, which enhance their functional performance in critical applications such as filtration [4,5], separation [6,7], and energy storage [8,9]. The hierarchical porosity of these membranes involves the integration of pores of different sizes (macro- (>50 nm), meso- (2–50 nm), and micropores (<2 nm)) within a single material [10]. This multiscale architecture is crucial because it allows for optimized fluid dynamics and enhanced surface area, which significantly improves the performance of the membranes [11,12]. Larger pores facilitate a rapid flow and easy access for larger particles, whereas smaller pores provide the necessary selectivity for retaining finer particles and molecules [13]. Recent advancements in fabrication techniques, such as electrospinning [14,15,16], phase separation [17,18,19,20], and templating methods [21,22,23,24], have enabled researchers to design and produce these hierarchically porous membranes with greater precision. These techniques allow the controlled manipulation of pore sizes and distributions, resulting in membranes that can be customized for specific applications. For instance, electrospinning combined with phase separation [25,26,27] has led to membranes that exhibit excellent mechanical strength and chemical resistance while maintaining high permeability and selectivity.

The application of hierarchically porous membranes in filtration and separation processes has gained considerable attention, particularly in environmental engineering and industrial processes [28]. These membranes efficiently remove contaminants from water and air, making them ideal for wastewater treatment and purification [29]. The ability to fine-tune the pore structure enables membranes to selectively separate various substances including oils, dyes, and salts, thus improving the efficiency of separation processes in the chemical industry [30]. Hierarchically porous membranes play a pivotal role in enhancing the performance of batteries and supercapacitors [31,32,33,34,35]. Their high surface area and tunable porosity contribute to an increased charge storage capacity and faster ion transport, leading to improved energy density and power output [36,37]. The efficiency and lifespan of energy-storage devices can be significantly enhanced by integrating these membranes into these devices, making them more viable for commercial applications.

In conclusion, the recent progress in HPPMs is a significant step forward in material science and engineering. With their complex multilevel porous structures, these membranes are poised to transform into various applications, particularly filtration, separation, and energy storage, ultimately contributing to more sustainable and efficient technologies in the future. This review highlights the recent advances in the preparation methods for hierarchically porous structures and their progress in electrochemical energy storage applications, with the goal of offering valuable in-sights and references for future research.

## 2. Fabrication Techniques

Advances in fabrication techniques, including electrospinning, phase separation, and template-assisted methods, have contributed significantly to the development of hierarchical porous structures with precisely tailored pore sizes and well-controlled pore distributions. Electrospinning allows the creation of nanofibers with high surface areas, which are ideal for enhancing the overall functionality of membranes [38,39]. Phase-separation techniques provide additional control over pore formation, enabling the creation of uniform interconnected pore networks that optimize mass transfer [40]. Template-assisted methods further refine this process, allowing the design of membranes with specific pore geometries and distributions [41]. These techniques facilitate the production of membranes with enhanced surface areas, controlled porosity, and high performance for various applications such as filtration, catalysis, and energy storage.

### 2.1. Electrospinning

Electrospinning is a versatile technique used to create nanofibrous mats with large surface areas and significant porosity [42]. By applying a high voltage to a polymer solution, electrospinning causes the polymer to eject from a needle in a fine stream, forming fibers that solidify as they travel through air. This method allows for precise control over the fiber morphology and is particularly useful for fabricating hierarchical porous structures with tunable pore sizes [43].

#### 2.1.1. Core–Shell Fibers

Coaxial spinneret is used to produce core–shell fibers, where the core and shell materials possess distinct properties, offering enhanced functionality within the resulting structure [44]. In this method, two polymer solutions are simultaneously electrospun through a dual nozzle, forming a fiber with a core–shell configuration. This setup allows the integration of diverse materials, which can improve mechanical strength, enhance stability, or introduce novel functionalities. This design is particularly beneficial for fabricating hierarchical porous structures because the core and shell materials can be selectively processed to achieve varying pore sizes in each region.

**Figure 1:**
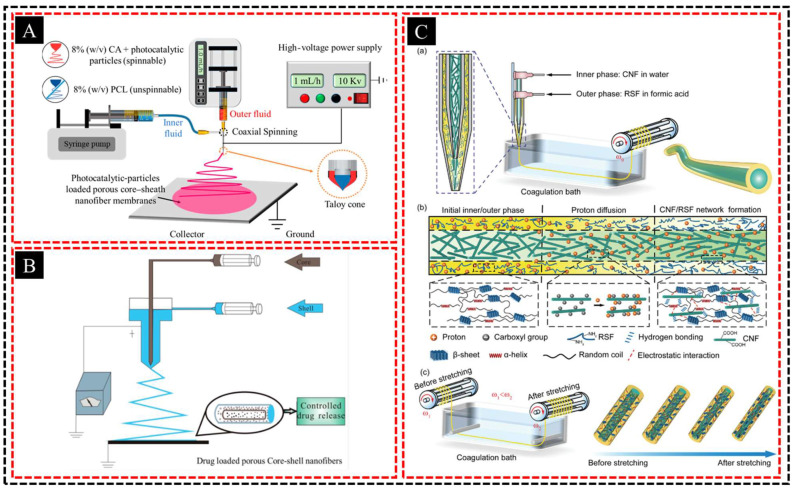
(**A**) Schematic diagram of the preparation process of electrospun nanofibers. (**B**) Schematic diagram of the coaxial electrospinning setup. (**C**) Design and fabrication of CNF/RSF core–shell fibers. (a) Preparation of core–shell fibers with a CNF core and an RSF shell using a co-axial microfluidic device via wet spinning. The coagulation bath consists of 60 vol% ethanol, 20 vol% glycerin, and 20 vol% water. (b) Schematics illustrating the non-covalent interactions within the core–shell fibers, including hydrogen bonding, van der Waals force, and electrostatic interaction. (c) Post-stretching of CNF/RSF fibers via two rotators rotating at two different angular velocities. After post-stretching, CNFs and RSFs are orientationally aligned along the fiber direction, facilitating the non-covalent interactions and enhancing the mechanical performances. Adapted with permission from Refs. [45,46,47]. Copyright 2024@MDPI. Copyright 2018@MDPI. Copyright 2023@Wiley.

For example, Yang et al. [45] successfully loaded AgCl/ZnO composite photocatalysts onto porous core–shell nanofibers composed of cellulose acetate (CA) and polycaprolactone (PCL) (Figure 1A). These functional fiber membranes demonstrated high efficiencies in the photocatalytic degradation of methylene blue, as confirmed by detailed electrochemical and degradation performance analyses. Similarly, Wang et al. [46] developed tea polyphenol (TPP)-loaded porous core–shell fibers using coaxial electrospinning and investigated the morphology, structure, and drug-release properties of the developed product (Figure 1B). The porous structure significantly enhanced the surface area and hydrophobicity, leading to improved TPP-release performance. Xiao et al. [47] created strong and tough hierarchical core–shell fibers using cellulose nanofibrils (CNFs) as the core and regenerated silk fibroins (RSFs) as the shell, thus producing spider silk (Figure 1C). These fibers exhibit remarkable mechanical properties, including high tensile strength and toughness, which make them ideal for advanced structural applications. Incorporating nanoparticles or functional groups into the polymer solution during electrospinning further enhances the fiber properties by introducing functionalities such as antimicrobial activity, catalytic effects, and electrical conductivity. When coupled with hierarchical pore generation strategies, these functionalized fibers exhibit not only specialized chemical and physical capabilities but also optimized structural features, further improving their performance in diverse applications.

#### 2.1.2. Controlled Morphology

**Figure 2:**
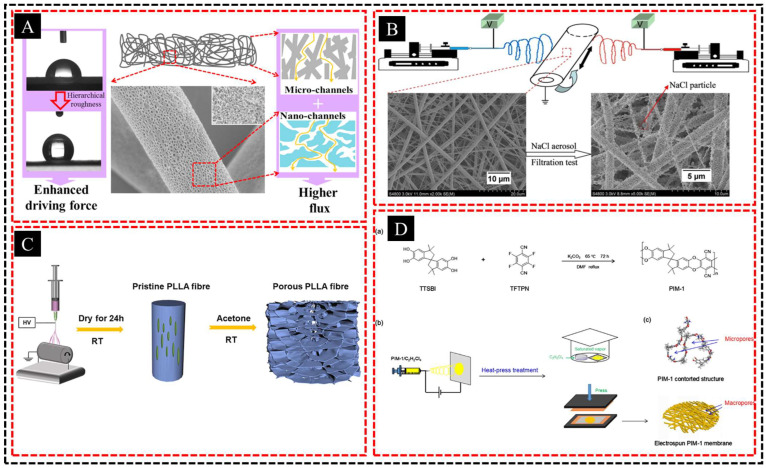
(**A**) Schematic illustration of double roles of hierarchically porous structures in the separation of oil/water emulsion. (**B**) Schematic diagram of the electrospinning apparatus. (**C**) Schematic illustration of fabrication of hierarchically porous PLLA fiber. (**D**) (a) The condensation polymerization reaction procedure for synthesis of PIM-1. (b) The fabrication of electrospun PIM-1 membrane. (c) The hierarchical porous structure of electrospun PIM-1 membrane. Adapted with permission from Refs. [48,49,50,51]. Copyright 2018@Elsevier Publisher, Copyright 2015@Elsevier Publisher, Copyright 2021@Wiley, Copyright 2018@MDPI.

Controlling the morphology of fibrous membranes is essential for the development of hierarchically porous structures with tailored properties. By fine-tuning the electrospinning parameters, such as the solution concentration, applied voltage, and distance between the needle and collector, researchers achieved precise control over the fiber diameter and morphology. The concentration of the polymer solution significantly influenced its viscosity and conductivity, which directly affected the fiber formation. Higher concentrations typically yielded thicker fibers, whereas lower concentrations produced finer fibers. By optimizing the solution concentration, a controlled range of fiber diameters can be obtained, which contributes to the desired pore structure within the membrane. For example, Zhao et al. [48] developed hierarchically porous poly (l-lactic acid) (PLLA) fabrics using electrospinning and water etching, achieving micro- and nanopores that enhanced gravity-driven oil/water separation (Figure 2A). The solution viscosity was controlled by adjusting the PLLA/PEO ratio, thereby tuning the fiber thickness and pore size. Similarly, Wang et al. [49] fabricated hierarchical PLA membranes by combining porous PLA fibers with PLA nanofibers in various configurations (Figure 2B). A double-layer PLA-N/PLA-P membrane with a 1/5 mass ratio showed a high filtration efficiency and a low pressure drop, meeting the HEPA filter standards.

The voltage applied during electrospinning plays a pivotal role in fiber elongation and stretching as it travels from the nozzle to the collector [50]. By adjusting the voltage, researchers can manipulate fiber formation dynamics, enabling the production of fibers with varied diameters and morphologies. Higher voltages typically result in thinner fibers owing to increased electrostatic forces, whereas lower voltages produce thicker and more stable fibers [51,52]. The distance between the electrospun needle and collector also affects the fiber morphology. A shorter distance enhances the fiber alignment and reduces the diameter owing to increased electrostatic attraction, whereas a longer distance tends to produce more chaotic arrangements and thicker fibers [53,54]. Fine-tuning this distance can result in the formation of a hierarchical pore structure that enhances the overall performance of the membrane. For example, Lu et al. [55] prepared porous PLLA fibers by either removing the pore-forming polymer (CAB) or inducing nonsolvent crystallization (Figure 2C). In one method, CAB was electrospun with PLLA and then removed, whereas in the other method, acetone immersion induced PLLA recrystallization, generating porous structures throughout the fibers. Pan et al. [56] fabricated a PIM-1 microfiber membrane by electrospinning after synthesizing PIM-1 via condensation polymerization (Figure 2D). The resulting membrane exhibited a hierarchical porous structure with a high surface area, achieving adsorption capacities of 0.084 and 0.804 mmol/g for carbendazim and phenol, respectively.

By carefully adjusting these processing parameters, hierarchically porous membranes with specific fiber diameters and morphological features can be fabricated. This precise control over fiber characteristics is key to optimizing membrane properties, such as permeability, selectivity, and mechanical strength, making them highly suitable for applications in filtration, separation, and energy storage.

### 2.2. Phase Separation

Phase separation is a widely used method for creating hierarchically porous structures in polymers by demixing a homogeneous polymer solution into distinct phases, followed by solidification [57,58]. This process enables the formation of macro-, meso-, and micropores within the material, each contributing to different functionalities and improving the overall performance of the resulting structure. In hierarchical-porosity preparation, phase separation typically involves techniques such as thermally induced phase separation (TIPS) [59] and nonsolvent-induced phase separation (NIPS) [60]. By controlling parameters such as the polymer concentration, solvent type, or temperature gradient, various pore sizes and distributions can be achieved, yielding a hierarchical network of pores.

#### 2.2.1. Thermally Induced Phase Separation (TIPS)

**Figure 3:**
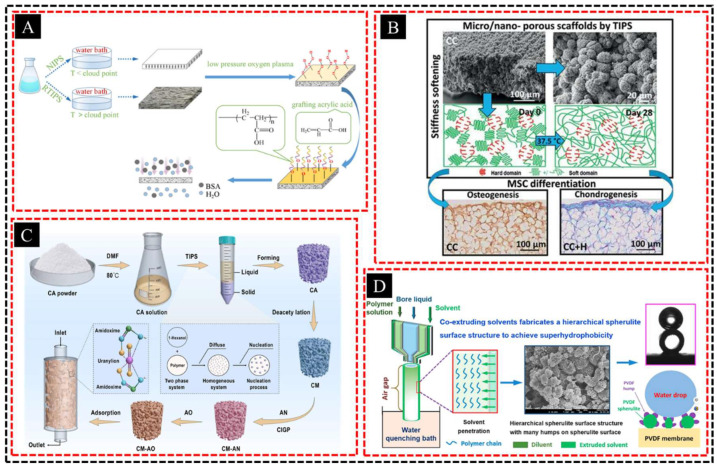
(**A**) Schematic diagram of the PES membrane preparation process. (**B**) Schematic illustration of hierarchically porous nanohybrid membranes promotes niches for mesenchymal stem cell differentiation. (**C**) Synthesis route of amidoxime cellulose monolith (CM-AO) and Dynamic column adsorption schematic diagram forming. (**D**) Hollow fiber membranes with hierarchical spherulite surface structure developed by thermally induced phase separation. Adapted with permission from Refs. [61,62,63,64]. Copyright 2020@Wiley, Copyright 2019@Wiley, Copyright 2024@ Elsevier Publisher, Copyright 2021@Elsevier Publisher.

TIPS is a fabrication technique in which a polymer solution is initially heated to fully dissolve the polymer, followed by gradual cooling to induce phase separation. The process begins by heating the polymer–solvent mixture above a critical temperature to ensure that the polymer is completely dissolved. As the solution cools, phase separation occurs, which typically results in the formation of polymer-rich and polymer-poor phases. While the polymer-rich phase solidifies, the polymer-poor phase, containing a solvent or nonsolvent, forms a porous network within the solidified structure. After phase separation, the solvent is typically removed by extraction or evaporation, leaving behind a porous material. Yang et al. [61] employed surface grafting modification combined with the reverse thermally induced phase separation (RTIPS) method to enhance the structure and permanent hydrophilicity of poly ether sulfone (PES) membranes with a hierarchical porous structure (Figure 3A). The modified membranes exhibited improved hydrophilicity and antifouling performance, achieving a maximum pure water flux of 1646.24 L/(m^2^·h) and a BSA rejection rate of 94.5%, with the hierarchical porosity contributing to a water flux recovery ratio exceeding 85% in long-term tests. In another case, Wu et al. [62] fabricated hierarchical porous membranes using a poly(urea–urethane) (PUU) nanohybrid through thermally induced phase separation (TIPS) at various temperatures (Figure 3B). The membranes exhibit thermoresponsive stiffness softening due to a phase transition from a semicrystalline to a rubbery state, characterized by the self-assembly of the quasi-random nanophase structure near the melting point of the crystalline domains of the soft segments at body temperature.

A key advantage of TIPS is its precise control over pore size and distribution. By adjusting parameters such as the cooling rate, polymer concentration, and solvent selection, the resulting pore structure can be finely tuned. Faster cooling rates tend to yield smaller and more uniform pores, whereas slower cooling rates produce larger and more interconnected pore networks. Moreover, TIPS enables the creation of porous membranes with a wide range of pore sizes, from nanometers to micrometers, making it highly versatile for various applications. For example, Sun et al. [63] introduced a novel hierarchical porous amidoxime cellulose monolith synthesized via TIPS (Figure 3C), demonstrating outstanding uranyl ion adsorption with a removal rate of 99.99% in batch tests and over 99.96% total α and β radioactivity removal in real wastewater. The strong mass transfer capability and recyclability of the monolith for at least five cycles underscore its potential for efficient nuclear wastewater treatment. Similarly, Fang et al. [64] developed polyvinylidene fluoride (PVDF) hollow fiber membranes using a one-step TIPS process with solvent co-extrusion, resulting in a porous structure that significantly in-creased water vapor permeability to 13.5 L m^−2^ h^−1^ (Figure 3D). The formation of surface spherulites, including novel hierarchical structures, created a superhydrophobic surface, greatly improving the wetting resistance of membranes for membrane distillation in low-surface-tension saline water applications. This level of control over pore structure makes TIPS an ideal method for creating membranes suited to diverse industrial needs, from water treatment to gas separation and energy storage.

TIPS is particularly beneficial for producing porous membranes for applications in filtration, chemical industry [65], and energy field [66]. The ability to control the porosity along with the mechanical strength of the material makes it suitable for applications where permeability, selectivity, and mechanical stability are critical. Additionally, TIPS is often favored owing to its simplicity, scalability, and environmental friendliness because it does not necessarily rely on toxic solvents or complex processing steps.

#### 2.2.2. Nonsolvent-Induced Phase Separation (NIPS)

**Figure 4:**
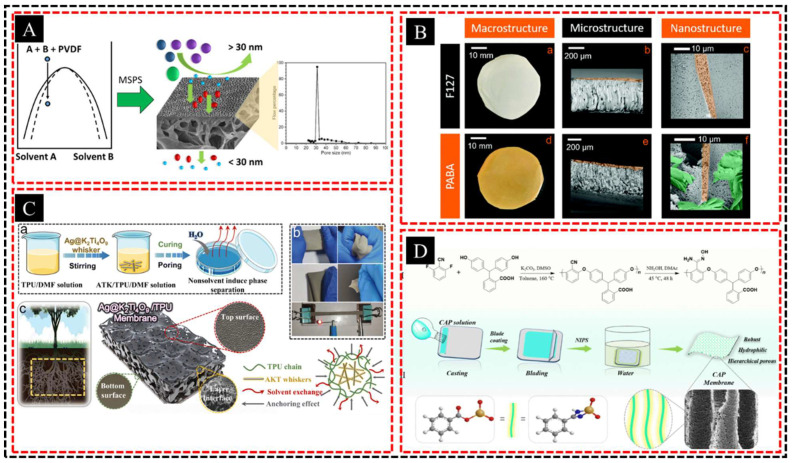
(**A**) Preparation of highly porous polymer membranes with hierarchical porous structures via spinodal decomposition of mixed solvents with UCST phase behavior. (**B**) Morphology of the macro-, micro- and nanostructures of Hierarchical Porous membrane fabricated via NIPS. (**C**) The fabrication process (a) and digital photos (b) of the TPU/AKT membrane. (c) Structure display and formation mechanism. (**D**) Fabrication of a flexible hierarchical porous CAP membrane. Synthesis scheme of CNP and CAP polymers. Adapted with permission from Refs. [67,68,69,70]. Copyright 2018@American Chemical Society, Copyright 2021@Royal Society of Chemistry, Copyright 2022@Royal Society of Chemistry, Copyright 2024@Royal Society of Chemistry.

NIPS involves demixing a homogeneous polymer solution into distinct phases, typically driven by changes in temperature, solvent composition, or concentration. As the system underwent demixing, regions of polymer-rich and polymer-poor phases formed. During solidification, the polymer-rich phase creates a solid framework, whereas the polymer-poor phase, which often contains a solvent or nonsolvent, forms pores. This technique allows the creation of porous structures with tunable properties, such as pore size and distribution, by adjusting factors such as polymer concentration, solvent selection, and the rate of phase separation. The resulting porous materials with their interconnected networks and high surface areas are highly desirable for applications in filtration membranes, tissue scaffolds, and separation technologies. Thankamony et al. [67] developed highly porous, asymmetric PVDF membranes using a combination of spinodal decomposition and NIPS (Figure 4A). This approach produces a mesoporous surface layer and a macrovoid-rich bulk layer, forming interconnected hierarchical porous structures that enhance permeability, selectivity, and mechanical strength. Similarly, Southern et al. [68] introduced hierarchical porosity into PES membranes via the NIPS method, with F127 controlling larger pores (10 μm) and PABA introducing finer structural hierarchy at the 1-μm scale to improve pore connectivity (Figure 4B). The dipolar interactions of PABA with PES, along with its orange color, facilitates the tracking of its removal and potential reuse, aligning with the principles of green chemistry. Additionally, Chen et al. [69] developed a breathable piezoresistive sensor using a hierarchical porous thermoplastic polyurethane (TPU)/Ag@K_2_Ti_4_O_9_ (AKT) hybrid membrane fabricated by NIPS (Figure 4C). The mem-brane asymmetric structure, with a graded pore size distribution due to AKT whiskers, resulted in high air permeability and sensitivity (0.127 kPa^−1^) across a wide working range (5 Pa–100 kPa), demonstrating its promise for sensor applications. Mi et al. [70] fabricated a hierarchical porous CAP membrane through nucleophilic polycondensation of phenolphthalein and 2,6-difluorobenzonitrile, followed by amidoximation and NIPS (Figure 4D). The strong π–π interactions of the aromatic monomers enhanced the mechanical properties and processability of the membrane in organic solvents. Through precise control of these processing parameters, NIPS enables the development of porous materials with tailored properties, making them highly versatile for a broad spectrum of applications.

### 2.3. Template-Assisted Methods

Template-assisted techniques are widely employed to create materials with precisely defined porous structures by using a template that determines the desired shape and porosity. This method is particularly effective for achieving multilevel porosity, where different pore sizes and distributions can be engineered to enhance the functionality in various applications.

#### 2.3.1. Nanotemplating

**Figure 5:**
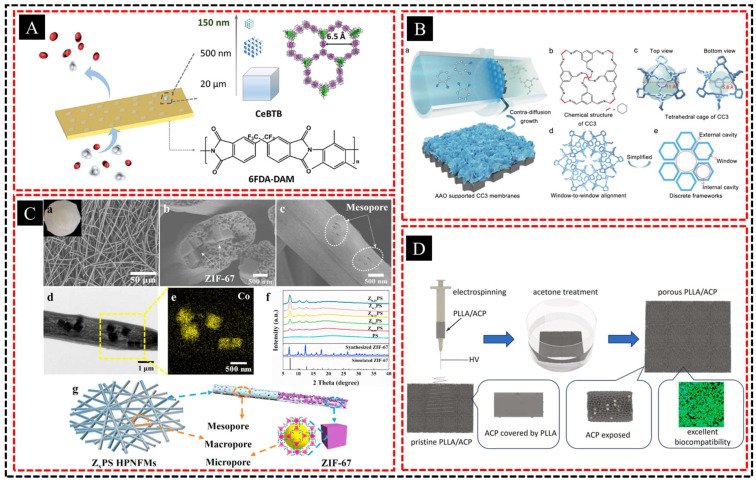
(**A**) Schematic illustration of fabricating a mixed matrix membrane with hierarchical supra-nanostructured porous coordination polymer filler. (**B**) CC3 membranes with hierarchical channels for ion transport. (a) Scheme of the fabrication of CC3 membranes via the contra-diffusion growth method. (b) Chemical structure of CC3. (c) Top and bottom views of the CC3 tetrahedral cage (hydrogen atoms are set as invisible for clarity). CC3 crystals consist of an internal tetrahedral cavity (indicated by a green sphere with a diameter of 11 Å) and four triangular windows (indicated by a green circle with a diameter of 5.8 Å). (d) Window-to-window alignment of CC3 cages. (e) Simplified model of CC3 channels composed of discrete frameworks. The subnanometer windows of CC3 can sieve ions for selective separation, and the internal cavities within CC3 together with the external cavities among CC3 can provide routes for fast ion transport. (**C**) (a) FESEM image of the surface of the Z0.2PS HPNFM, and the inset is a digital photograph of the membrane, (b,c) FESEM image of the cross-section and surface of the Z0.2PS HPNFM, (d) TEM image of the Z0.2PS HPNFM, (e) EDX mapping of the element Co, (f) XRD patterns of the ZxPS HPNFMs with various ZIF-67 contents, and (g) schematic of the hierarchical porous structure of the ZxPS HPNFMs. (**D**) Schematic illustration of the fabrication of Hierarchical porous PLLA/ACP fibrous membrane. Adapted with permission from Refs. [71,72,73,74]. Copyright 2020@Elsevier Publisher. Copyright 2022@American Chemical Society, Copyright 2020@Elsevier Publisher, Copyright 2024@Elsevier Publisher.

Nanotemplating, particularly using anodic aluminum oxide (AAO) templates, plays a crucial role in creating structures with highly controlled pore sizes at the nanoscale level. These templates are used to synthesize materials with hierarchical porosity, often referred to as multilevel or multiscale pores. The concept of hierarchical pores involves the formation of materials that contain pores of varying sizes, typically micro-, meso-, and macro-pores distributed throughout the structure. This multiscale porosity enhances the surface area, mass transport, and catalytic activity of the material. Zhou et al. [71] focused on creating mixed-matrix membranes (MMMs) for enhanced CO_2_/CH_4_ separation by incorporating a chemically stable PCP filler, CeBTB, which features a one-dimensional open channel of 6.5 Å (Figure 5A). The incorporation of a hierarchical supra-nanostructured CeBTB filler (150 nm) into the porous 6FDA-DAM polymer matrix facilitated gas diffusion, resulting in improved CO_2_ permeability, enhanced CO_2_/CH_4_ selectivity, and reduced mem-brane plasticization.

Hierarchical pores can be prepared using AAO templates via various deposition methods, such as electrochemical deposition or sol-gel processes. The small uniform pores created by AAO templates serve as scaffolds, allowing for the growth of nanostructures with controlled pore dimensions. By modifying the template or deposition conditions, the pore size and distribution can be tailored. For example, Xu et al. [72] developed porous organic cage (CC3) membranes featuring hierarchical channels with internal and external cavities connected by sub-nanometer-sized windows that selectively sieve monovalent ions from divalent ones (Figure 5B). The membranes were fabricated via contra-diffusion growth on AAO substrates, allowing fast ion transport through internal and external cavities.

Furthermore, the integration of different pore sizes within a material enhances its performance in various applications. For instance, macropores facilitate rapid diffusion of molecules, whereas smaller mesopores and micropores increase the surface area for reactions or adsorption. Hu et al. [73] developed a zeolitic imidazolate framework (ZIF-67)-trap-in polystyrene hierarchical porous nanofiber membrane (ZIF-67/PS HPNFM) for efficient PM and SO_2_ filtration, featuring a hierarchical porous structure that minimizes resistance (Figure 5C). The interconnected meso- and macro-porous structures of the PS nanofiber membrane enhanced gas diffusion, whereas the ZIF-67 loading increased the surface area and microporosity, significantly improving SO_2_ adsorption. Meng et al. [74] incorporated amorphous calcium phosphate (ACP) nanoparticles into PLLA to create electrospun PLLA/ACP fibrous membranes that were treated with acetone to achieve a hierarchical porous structure with an ultrahigh surface area (Figure 5D). Acetone treatment exposed the ACP nanoparticles on the fiber surface, resulting in superior mechanical properties and enhanced wettability. This hierarchical pore structure is particularly advantageous for catalysis, filtration, drug delivery, and energy storage devices, such as batteries and supercapacitors.

#### 2.3.2. Self-Assembly

Self-assembly is a powerful and elegant technique to create hierarchical porous structures. This relies on the spontaneous organization of molecules, particularly polymers, into well-defined and ordered architectures without external intervention. By harnessing this natural tendency, complex porous networks with tunable properties can be designed. A commonly used approach involves the use of block copolymers or surfactants as structure-directing agents.

**Figure 6:**
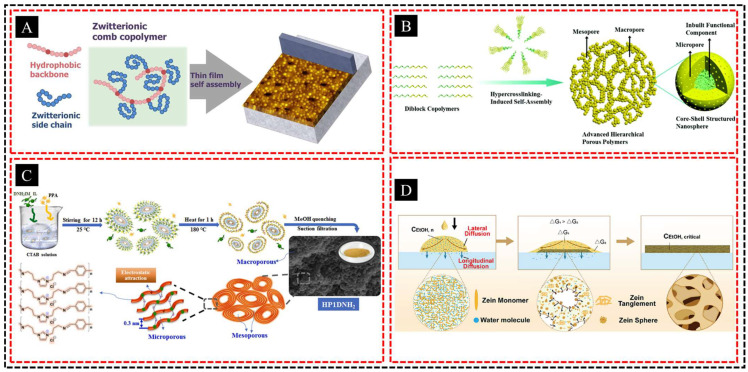
(**A**) Schematic illustration of porous, thin films with hierarchical structures formed by self-assembly of zwitterionic comb copolymers. (**B**) Schematic illustration of the synthesis of advanced hierarchical porous polymeric materials by hypercrosslinking-induced self-assembly. (**C**) Preparation of ionic liquid covalent organic polymers HP_1_DNH_2_. (**D**) Schematic illustration of the dynamic porous structure formation process that involved the longitudinal diffusion of ethanol, the laterally diffuse aggregation of zein molecules, and the formation of the porous film matrix. CEtOH, n and CEtOH, critical mean the initial concentration and critical concentration of ethanol, respectively. ΔG = CEtOH, n – CEtOH, critical. Adapted with permission from Refs. [75,76,77,78]. Copyright 2023@Elsevier Publisher, Copyright 2017@ Royal Society of Chemistry, Copyright 2023@Elsevier Publisher, Copyright 2022@American Chemical Society.

In this method, block copolymers or surfactants form micelles or other organized patterns when dispersed in a solvent. These structures act as templates for the deposition of inorganic and organic materials. As the solvent evaporates or the environmental conditions change, the organized polymer or surfactant patterns guide the formation of the surrounding material, resulting in porous networks. Subsequently, the polymer or surfactant is removed, leaving behind a well-defined porous structure. Pore size and morphology can be precisely controlled by adjusting the size, composition, or ratio of the self-assembled components. This self-assembly technique is highly versatile and scalable, enabling the production of multiscale or hierarchical pores that enhance the material performance across various applications. For example, Kaner et al. [75] explored the self-assembly of zwitterionic comb-shaped copolymers (ZCCs) with varying side-chain densities and lengths, which formed different morphologies depending on the film preparation method (Figure 6A). When applied to porous substrates via phase separation, ZCCs produce hierarchical features, including spherical micelles and nanopores, which influence the water permeance and morphology, particularly in saline environments. Similarly, Xu et al. [76] developed a hypercrosslinking-induced self-assembly strategy to fabricate 3D interconnected hierarchical porous polymers (AHPPs) using diblock copolymers (Figure 6B). This method enables the precise control of the nanostructure, leading to significantly improved adsorption properties for phenol and CO_2_. Wang et al. [77] employed a rapid CTAB-assisted evaporation-induced self-assembly method to synthesize flexible hierarchical porous covalent organic polymers (HPnDNH_2_) within 1 h (Figure 6C). The resulting ordered porous structure exhibited significantly enhanced efficiency in removing ReO^4−^/TcO^4−^. Cai et al. [78] developed a rapid method to fabricate biopolymer-based hierarchically porous films within 5 s by inducing interfacial self-assembly of prolamins at the air-liquid interface during antisolvent dripping (Figure 6D). These films featured location-graded Janus structures with tunable porosities and pore sizes that were controlled by the solvent gradients and operating conditions.

Another advantage of self-assembly is the flexibility in the choice of materials. It is compatible with a wide range of materials, including metals, oxides, and polymers, making it suitable for numerous applications in various fields, such as biomaterials, filtration, sensors, and nanotechnology. The ability to design hierarchical porous structures via self-assembly also offers significant potential for sustainable and green chemistry, as it reduces the need for harsh processing conditions or toxic chemicals.

#### 2.3.3. Crystallization Templates

The use of crystallization templates is an advanced and highly effective technique for creating hierarchical porous structures, particularly polymers [79]. This method harnesses the natural crystallization process of materials such as polymers or inorganic compounds to form templates that guide the development of intricate porous architectures [80]. Controlled crystallization of a specific material often results in well-defined, ordered structures that can act as a mold or scaffold for the formation of a porous network [81].

In the crystallization template approach, the crystalline regions of a material serve as a framework around which other substances are deposited. After deposition, the crystal-line template can be selectively removed via thermal decomposition, chemical dissolution, or other processes, leaving behind a porous structure. The resulting pores mirror the morphology and size of the initial crystalline regions, enabling the creation of complex interconnected porous networks. This method offers precise control over pore size and distribution, with the added benefit of hierarchical organization, meaning that pores of different sizes, ranging from micro- to meso- and macro-pores, can coexist within the same structure. A key advantage of using crystallization templates is their ability to tailor porous structures for specific applications. For example, in polymer-based systems, crystallization templates can generate porous materials with enhanced mechanical strength, thermal stability, and chemical resistance. These materials are particularly useful for filtration, engineering, and energy storage applications.

**Figure 7:**
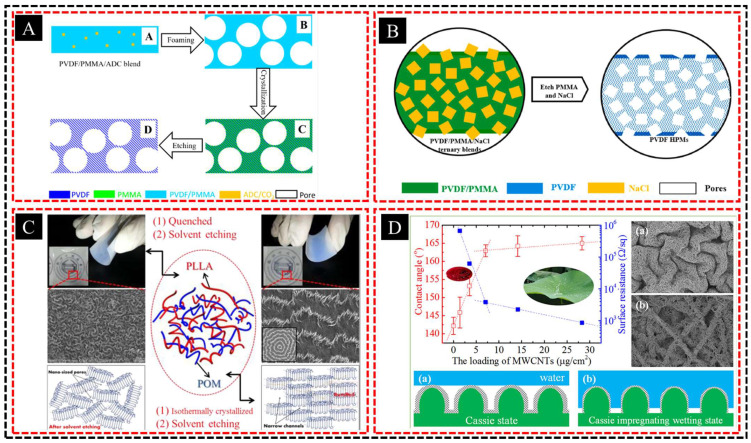
(**A**) Illustration of fabrication of PVDF HPMs via the combination of crystallization template and foaming. The specimen of hot-pressed PVDF/PMMA/ADC blend (A), upon foaming (B), isothermal crystallization (C) and etching (D). (**B**) The schematic illustration of PVDF HPMs prepared in PVDF/PMMA/NaCl blends with highly filled NaCl. (**C**) Schematic Illustration of the “Crystallization-Modulated” 3D Interpenetrated Nanoporous POM Materialsa. (**D**) The MWCNTs act as nanoscale structures, creating hierarchical surface roughness. SEM images of the top surface of the POM nonwovens with the indicated MWCNT loadings. a: 28.29 μg/cm^2^, b: 3.54 μg/cm^2^ (a) Slippery superhydrophobicity, where the droplet is excluded from both micro- and nanoscale structures. (b) Sticky superhydrophobicity (Cassie-impregnating wetting state), where the microscale structures are partially wetted by the droplet whereas the nanoscale structures are inaccessible to the droplet. The protrusions represent the surface fluctuation provided by POM fibers, and the black lines (or meshes) are MWCNTs. Adapted with permission from Refs. [83,84,85,86]. Copyright 2022@MDPI, Copyright 2023@Elsevier Publisher, Copyright 2015@American Chemical Society, Copyright 2017@American Chemical Society.

For example, Lin et al. [82] studied PMMA exclusion behavior during PVDF crystallization in a PVDF/PMMA blend system. Hierarchically porous PVDF membranes with isolated large pores and continuous narrow nanopores were then fabricated using a blend of PMMA by combining crystallization templating with chemical or supercritical CO_2_ foaming [83] (Figure 7A). The optimal membrane produced using a chemical foaming agent exhibited a significantly higher permeability (up to 20 times) without losing selectivity, which was attributed to the large pores facilitating diffusion and the nanopores maintaining selectivity. Furthermore, they demonstrated that hierarchically porous PVDF membranes with cubic-large-pores connected by narrow nanopores could be fabricated from a PVDF/PMMA/NaCl blend [84] (Figure 7B), exhibited up to 200 times higher permeability without loss of selectivity due to the synergistic effects of large internal and surface pores, which enhance diffusion and separation efficiency.

Crystallization templating can be used to produce highly uniform and reproducible porous structures. By carefully controlling the crystallization conditions, such as temperature, concentration, and solvent type, it is possible to fine-tune the size and morphology of the resulting porous network. Ye et al. [85] developed nanoporous poly(oxymethylene) (POM) materials with hierarchically patterned surfaces and 3D interpenetrated channels by blending POM with PLLA and extracting the amorphous PLLA phase (Figure 7C). The co-continuous structure formed by the POM lamellae and expelled PLLA can be tailored by adjusting the crystallization conditions, allowing control the pore size and morphology. Overall, crystallization templating offers sophisticated and customizable pathways for producing hierarchical porous materials with high precision, making them invaluable tools for advanced material design and manufacturing. Further, Ye et al. [86] developed a superhydrophobic, electrically conductive POM nonwoven with a two-tier micro- and nanoporous structure using PLLA/POM-blended nonwovens and by filtering multiwalled carbon nanotube (MWCNT) suspensions (Figure 7D). The “vine-on-fence” and cerebral cortex-like surfaces, with tunable conductivity and water adhesion, were created by adjusting the MWCNT loading, offering a versatile method for fabricating functional super-hydrophobic surfaces.

### 2.4. Combination Techniques

The combination of different fabrication techniques offers a powerful approach for significantly enhancing the properties of membranes and tailoring them for specific ap-plications. By merging complementary methods, it is possible to create membranes with optimized performance characteristics such as improved permeability, selectivity, mechanical strength, and chemical resistance.

#### 2.4.1. Electrospinning + Phase Separation

**Figure 8:**
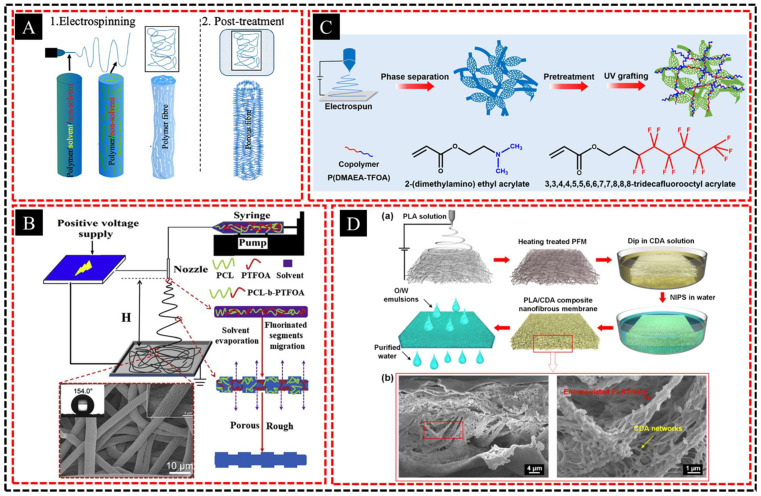
(**A**) Structures of each stage of the electrospinning and the post-treatment process. (**B**) Schematic diagram of the electrospinning nanofiber membrane. (**C**) Schematic Illustrating the Fabrication of a hierarchical beadlike porous PS fibrous membrane with pH-switchable wettability. (**D**) (a) Schematically illustrating the preparation process and (b) cross sectional SEM images of the PLA/CDA composite nanofibrous membrane for oil-in-water emulsions separation. Adapted with permission from Refs. [87,88,89,90]. Copyright 2019@American Chemical Society, Copyright 2020@Elsevier Publisher, Copyright 2020@American Chemical Society, Copyright 2024@Elsevier Publisher.

Electrospun fibers can be embedded in a matrix formed by phase separation to create a composite material with hierarchical porosity and enhanced mechanical properties. The combination of electrospinning and phase separation is particularly effective for fabricating hierarchical porous structures because it allows precise control over the pore-size distribution while simultaneously improving the mechanical strength of the composite. Electrospun fibers are typically created from polymers using an electrospinning process that produces ultrafine fibers with diameters ranging from nanometers to micrometers. These fibers have a large surface area and can be arranged in a network, which contributes to the mechanical reinforcement of the composite. When these fibers are embedded in a matrix, they act as a structural framework providing increased tensile strength, flexibility, and resilience. A PLLA nanofibrous membrane with an ultrahigh specific surface area was developed by Song et al. [87] using electrospinning and acetone post-treatment (Figure 8A). A porous structure was achieved by the nonsolvent-induced spinodal phase separation during electrospinning and sol-vent-induced recrystallization during post-treatment. The hierarchical porous structure and large surface area contributed to its superior performance, achieving a 99.99% filtration efficiency for ultrafine aerosols (30–100 nm).

Phase separation is a widely used technique to generate porosity within a material. This process involves the separation of two or more incompatible phases in a polymer blend, resulting in pore formation. The size and distribution of these pores can be controlled by manipulating factors such as temperature, solvent composition, and polymer concentration. When phase separation is applied in the presence of electrospun fibers, a matrix is formed around the fibers, creating a composite material with multiscale pores in which macropores, mesopores, and micropores coexist. Li et al. [88] developed a highly stretchable, biodegradable, superamphiphobic nanofibrous membrane with a hierarchical porous surface by electrospinning fluorinated polycaprolactone (PCL-b-PTFOA) (Figure 8B). Phase separation and fluorinated segment migration resulted in the formation of a hierarchical porous surface and enhanced the superhydrophobicity and mechanical properties of the membrane.

The preparation of hierarchical pores using this method has several advantages. First, large macropores, which are often formed through phase separation, provide efficient channels for fluid or gas transport and reduce the diffusion barrier. Smaller mesopores and micropores, which are often influenced by the electrospun fiber network, contribute to a high surface area, which is beneficial for applications requiring high selectivity, such as filtration, catalysis, and adsorption. Wang et al. [89] fabricated a hierarchical porous PS fibrous membrane with pH-switchable wettability via nonsolvent-induced phase separation during electrospinning, which enhanced the surface roughness and superwettability (Figure 8C). The membrane efficiently separates oil/water mixtures, achieving an oil flux of 10,186.8 L m^2^ h^−1^ and a separation efficiency of 99.2% under gravity.

Furthermore, the embedded electrospun fibers not only enhanced the mechanical properties of the matrix, but also contributed to the formation of smaller pores. The inter-play between the electrospun fibers and phase-separated matrix creates a synergistic effect, improving the overall stability, permeability, and functionality of the material. The result is a hierarchical porous composite with tunable properties that is ideal for applications such as water filtration, membrane separation, tissue engineering scaffolds, and energy storage systems such as batteries and supercapacitors. Jiang et al. [90] developed a PLA/cellulose diacetate (CDA) composite nanofibrous membrane featuring a unique hierarchical porous structure created by the in situ construction of hydrophilic CDA networks through a combination of electrospinning and nonsolvent-induced phase separation technologies (Figure 8D). This enhancement in hydrophilicity and underwater superoleophobicity enables the efficient separation of oil-in-water emulsions under gravity. In addition, the biodegradability and reusability of the membrane render it an environmentally friendly option for treating oily wastewater.

By adjusting parameters, such as the polymer type, fiber diameter, and phase separation conditions, it is possible to fine-tune the hierarchical porous structure to meet specific application requirements, making this method a versatile and efficient approach for fabricating advanced materials with hierarchical porous structures.

#### 2.4.2. Template Assisted + Electrospinning

**Figure 9:**
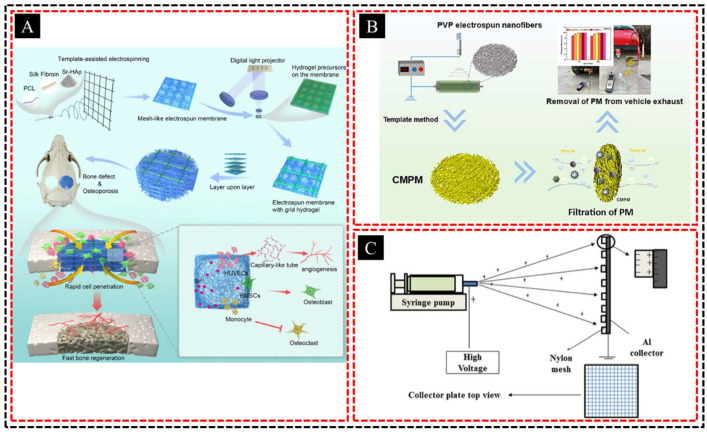
(**A**) Schematic overview of the research. Utilizing PCL, SF, and Sr-HAp as electrospinning materials, mesh-like electrospun membranes were fabricated through a template-assisted electrospinning technique, followed by the attachment of a grid hydrogel layer onto the membrane using the DLP methodology. Subsequently, these membrane layers were stacked to construct 3D scaffolds. These scaffolds were implanted into the cranial defects of OVX osteoporotic rats. The porous, extracellular matrix-mimicking structure facilitates rapid cellular infiltration and proliferation, while the microenvironment of the scaffold promotes angiogenesis and osteogenic differentiation, concurrently inhibiting osteoclast differentiation. This culminates in the fast regeneration of bone (**B**) Schematic Illustrating of the microporous polymer membranes prepared using PVP electrospun nanofibers as a template for efficient PM capture. (**C**) Schematic of the electrospinning setup used for the production of the three dimensional micropatterned nanofabric surfaces. Adapted with permission from Refs. [91,92,93]. Copyright 2024@ Springer Publisher, Copyright 2023@Elsevier Publisher, Copyright 2017@Wiley, Copyright 2022@Elsevier Publisher.

Template-assisted electrospinning is a powerful technique for fabricating hierarchical porous membranes with controlled pore sizes and interconnected networks. It combines templating methods, which use sacrificial particles or molds, with electrospinning to achieve highly ordered porous structures within the membrane. This approach allows for the precise tuning of membrane porosity, ensuring a uniform distribution of pores ranging from the micro- to nanoscale. The resulting hierarchical structure enhances both flux and selectivity, making these membranes ideal for advanced filtration and separation processes such as water purification, gas separation, and biomolecule filtration. The hierarchical porous architecture not only increases the surface area of the membrane, enabling higher throughput, but also promotes efficient transport through interconnected channels, reduces fouling, and improves long-term performance. Additionally, the flexibility of the electrospinning process allows the incorporation of various functional materials, further enhancing the chemical, thermal, and mechanical properties of the membrane. Thus, template-assisted electrospun membranes are highly versatile and can be tailored to specific industrial and environmental applications. Xiao et al. [91] (Figure 9A) developed a 3D scaffold with an in situ cured hydrogel and mesh-like hierarchical porous nanofiber membranes for enhanced mechanical strength and rapid cell infiltration. The Sr-HAp-enriched nanofibers enabled controlled Sr ion release, promoted angiogenesis and osteogenesis, and inhibited osteoclast differentiation. Yang et al. [92] developed conjugated microporous polymer membranes (CMPM) with a hierarchical porous structure using electrospun PVP nanofibers as a template (Figure 9B). These membranes feature macropores from nanofibers and mesopores from polymer synthesis, enabling the effective removal of particulate matter from air and vehicle exhaust. Kakunuri et al. [93] fabricated large-area, 3D micropatterned nanofibrous mats with a hierarchical porous structure using nylon mesh templates with varied opening sizes (50–200 μm) and cellulose acetate (CA) as the polymer precursor (Figure 9C). The tunable wettability of the CA nanofibers, influenced by the mesh size, enabled control over the solid–liquid–air interface and capillary pressure, creating versatile free-standing fabrics.

#### 2.4.3. Template Assisted + Phase Separation

The preparation of hierarchical porous membranes through a combination of crystallization templates and phase separation of incompatible systems is a versatile and innovative approach for membrane fabrication. This method involves leveraging two key processes—crystallization templating and phase separation—to create membranes with pores of varying sizes. Crystallization of templates is the first step in this method. As a polymer crystallizes, certain regions of the material form highly ordered crystalline structures, whereas other regions remain amorphous. These crystalline domains act as scaffolds or templates that direct the formation of porous networks. The size and distribution of the resulting pores are influenced by the nature of the polymer, rate of crystallization, and processing conditions, allowing the creation of pores in the nanometer-to-micrometer range. In a previous study [94], we fabricated PEEK porous membranes using crystallization templates in a miscible blend with PEI and selective etching. PEEK maintains excellent thermal and chemical stability owing to the melt-processing method. These membranes demonstrated long-term stability at 260 °C for particulate matter capture and effectively separated emulsions containing corrosive components and organic solvents, making them highly suitable for harsh environmental conditions.

**Figure 10:**
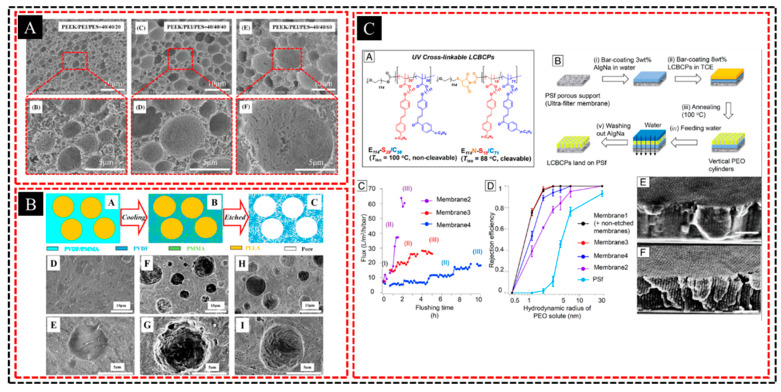
(**A**) Cross-sectional scanning electron microscope (SEM) images of hierarchically PEEK porous membranes with different ratios of PES at different magnifications. (A,B) PEEK/PEI/PES = 40/40/20, (C,D) PEEK/PEI/PES = 40/40/40, and (E,F) PEEK/PEI/PES = 40/40/60. (**B**) Schematic representation of formation mechanism of HPMs from PVDF/PMMA/PLLA blend upon extraction (A) to (C). (D) to (I) show SEM images of PVDF/PMMA/PLLA = 40/40/20 with lower (D,F,H) and higher (E,H,I) magnifications. Images (D,E), (F,G) and (H,I) represent original fracture, fracture immersed in sodium hydroxide solution and residual fracture washed by chloroform, respectively. (**C**) Bilayer membranes with asymmetric transporting channels derived from STAMPS breaking a constraint of tradeoff relationship between water flux and rejection efficiency. (A) Chemical structures of cross-linkable LCBCPs. (B) LCBCP direct-coating process on porous support. (i) Bar-coating AlgNa aqueous solution on PSf UF support. (ii) Bar-coating LCBCP solution. (iii) Annealing and subsequently cross-linking by UV irradiation (290 to 340 nm, 4 mW/cm^2^, 20 min). (iv and v) Flushing water at 40 °C to wash out AlgNa. LCBCPs land on the PSf support. (C) Increasing flux of LCBCP membranes by the NHS-ester cleavage. Each curve represents one of the following membranes: “Membrane2” (purple), “Membrane3” (red), and “Membrane4” (blue). At each stage of (I) to (III), water (I), 300 mM 2-aminoethanol aqueous solution (II), and water (III) were flushed. (D) Rejection efficiencies for various molecular weights of PEO solute (0.4, 2, 5, 11, 20, 44, 580 kg/mol). Each curve represents one of the following membranes: “Membrane1” (black) (all of the membranes before NHS-ester cleavage also exhibited the identical rejection efficiency to this curve), “Membrane3” (red), “Membrane4” (blue), “Membrane2” (purple), and PSf support (teal). The error bars represent the SD of three attempts. (E and F) Cross-sectional image of E114N-S18/C71 film before and after NHS cleavage on a silicon wafer.

Simultaneously, phase separation in incompatible polymer blend systems plays a crucial role in generating additional porosity. Incompatible polymer systems in which the components do not mix homogeneously tend to undergo phase separation during processing. This led to the formation of distinct polymer-rich and polymer-poor regions. By carefully controlling the phase separation process, these regions can be tailored to create a hierarchical pore structure with large macropores in polymer-poor regions and smaller meso- or micropores in polymer-rich regions. Finally, one of the polymer phases is selectively removed through solvent etching or thermal degradation, leaving behind a porous membrane. Combining the two processes—crystallization templating and phase separation—results in membranes with multiscale porosity and interconnected pores of different sizes, which enhance both permeability and selectivity. We then introduce a third component of PES into the PEEK/PEI blend system [95]. Thus, we developed hierarchically porous polyether ether ketone (PEEK) membranes (HPMs) by precisely manipulating a polymer ternary blend system to achieve a controlled pore morphology at two scales (Figure 10A). Finite element simulations confirmed that the hierarchical structure enhanced the separation efficiency, aligning closely with the experimental results. In another study [96], we fabricated a hierarchically porous PVDF membrane using a ternary PVDF/PMMA/PLLA blend (Figure 10B). PVDF served as the framework of the crystallization template; PMMA was expelled into the interlamellar region of the PVDF crystals and then removed, forming nanoscale narrow channels; and PLLA formed micron-scale pores owing to the phase separation of the incompatible system.

The hierarchical porous structure offers significant advantages, including improved mass transport owing to large macropores and enhanced surface area provided by smaller mesopores and micropores. This combination is particularly beneficial in separation processes, such as gas separation, water treatment, and membrane distillation, where both high flux and selectivity are essential. For example, Hibi et al. [97] developed a self-template-assisted microphase segregation (STAMPS) strategy to independently control the in-plane and out-of-plane self-assemblies in blended LCBCP films, enabling the formation of multilayered 3D mesostructures with varied morphologies (Figure 10C). This approach facilitates the design of hierarchical porous structures and enhances the separation performance by breaking the tradeoff between flux and rejection efficiency in permeable membranes. Moreover, the flexibility of this method allows the customization of pore sizes and membrane properties by adjusting the polymer composition, crystallization conditions, and phase separation dynamics. It also enables the use of a wide range of materials including polymers, inorganic compounds, and hybrid systems, making it suitable for diverse applications in biotechnology, environmental engineering, and energy storage.

These advancements in fabrication techniques not only improve the performance of hierarchically porous membranes but also enable customizations for specific applications. Each method provides unique advantages, and their selection often depends on the desired characteristics of the final membrane.

## 3. Application of Hierarchically Polymer Membranes in Next Generation Electrochemical Energy Storage Devices

HPPMs are emerging as key components in the development of next-generation electrochemical energy storage devices [98,99] owing to their unique and advantageous structural characteristics. These membranes, which feature a multiscale pore structure comprising both micropores (pores smaller than 2 nm) and mesopores (pores ranging from 2 to 50 nm), were engineered to optimize several critical parameters for energy storage applications. The combination of high surface area, efficient ion transport pathways, and superior mechanical stability makes these materials particularly promising for use in advanced technologies, such as lithium-ion batteries, supercapacitors, and other novel energy storage systems [100,101]. A primary advantage of HPPMs is their ability to significantly enhance ion transport. The interconnected pore networks allow the efficient movement of ions through the membrane, reducing the resistance encountered during the charge and discharge processes. This leads to faster electrochemical reactions and improved rate capabilities, which are crucial for high-performance batteries and supercapacitors. In addition, multiscale porosity provides ample surface area for ion adsorption and storage, contributing to higher energy densities and greater charge storage capacity. Improved electrolyte accessibility is another important benefit of these membranes. The hierarchically porous structure ensures that the electrolyte can easily permeate the material, enhance ionic conductivity, and promote uniform ion distribution across the electrode surfaces [102]. This feature is particularly important in high-energy-density devices, where homogeneous interactions between the electrolyte and electrode material are essential for achieving stable and efficient performance. By facilitating better electrolyte accessibility, HPPMs can help overcome the limitations associated with the traditional dense or poorly porous separators used in conventional batteries. Mechanical stability is also a key factor for these membranes to excel. Their robust architectures, which combine micro- and mesoporous structures, provide superior mechanical strength and flexibility [103]. This is particularly valuable in the context of long-term cycling stability, wherein materials are subjected to repeated charge/discharge cycles, leading to mechanical degradation. Hierarchically porous membranes can withstand the stresses associated with volumetric expansion and contraction during operation, ensuring the longevity and reliability of energy storage devices. The versatility of HPPMs further enhances their applicability in a wide range of energy-storage technologies.

### 3.1. Batteries

In rechargeable batteries, especially lithium-ion batteries (LIBs), sodium-ion batteries (SIBs), and lithium–sulfur (Li-S) batteries, hierarchically porous membranes are critical because of their large surface area, tunable pore size, and enhanced electrical conductivity [104,105,106] The hierarchical pore structure allows more lithium ions to interact with the electrode, thereby improving the capacity and rate performance. This is crucial for fast charge-discharge cycles. The interconnected pores provide efficient pathways for ion transport, reducing the internal resistance and improving electrochemical performance. The enhanced mechanical stability and flexibility of these membranes prevent dendrite growth, which is a major issue in lithium-metal batteries, thereby improving their safety and cycle life [107]. For Li (Li-S) batteries, the high surface area of hierarchically porous membranes can physically trap soluble polysulfides, minimizing the shuttle effect (in which polysulfides dissolve in the electrolyte and reduce battery efficiency) [108,109]. The conductive network in these membranes enhances sulfur utilization and boosts over-all battery performance.

#### 3.1.1. Separators

**Figure 11:**
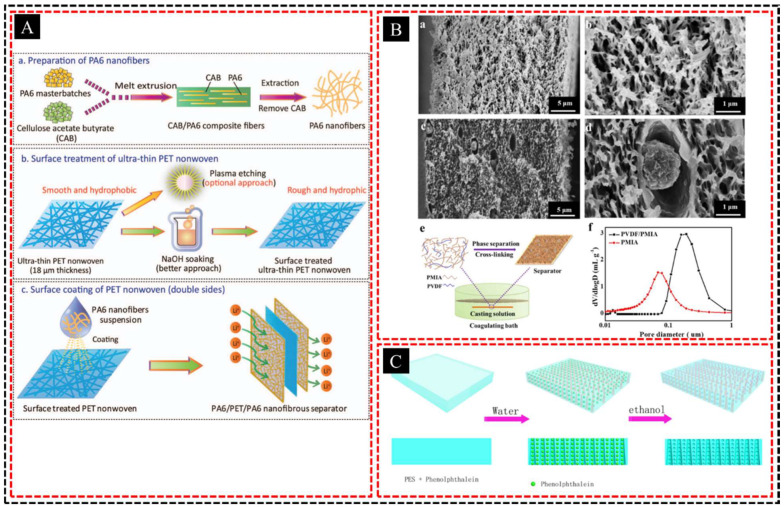
(**A**) Schematical illustration of the preparation process of PA6/PET/PA6 nanofibrous separator. (**B**) SEM images of (a,b) pure PMIA separator and (c,d) PVDF/PMIA blended separator, (e) phase transformation mechanism scheme, and (f) pore size distribution of pure PMIA separator and PVDF/PMIA blended separator. (**C**) Design and fabrication of hierarchical porous membranes (polyethersulfone was used as the starting materials and phenolphthalein was used as template). Adapted with permission from Refs. [110,111,112]. Copyright 2017@ECS, Copyright 2021@ECS, Copyright 2017@ Elsevier Publisher.

HPPMs can significantly enhance the performance of LIBs by improving ion transport and increasing the contact area between the electrolyte and electrode materials. The multiscale porosity allows for better electrolyte retention and distribution, leading to higher ionic conductivity and faster charging/discharging rates. Incorporating these membranes as separators in LIBs can reduce the internal resistance and prevent short circuits while maintaining structural stability, even at elevated temperatures. This is particularly relevant for high-power applications where traditional separators may not offer sufficient ion conductivity or thermal stability. In addition, the porous structure can help mitigate issues such as lithium dendrite formation, thereby improving the safety and lifespan of batteries. Chen et al. [110] developed a hierarchically structured polyamide 6 (PA6) nanofibrous separator (PA6/PET/PA6) using a poly (ethylene terephthalate) (PET) nonwoven substrate treated with NaOH for better adhesion (Figure 11A). This separator demonstrated lower thermal shrinkage, higher electrolyte affinity, and better cycling performance than commercial polypropylene separators, making it a promising choice for high-rate lithium-ion batteries. Li et al. [111] fabricated PVDF/PMIA-blended separators with hierarchical porous structures, offering enhanced electrolyte absorption and retention, as well as improved wettability, thermal stability, and ionic conductivity compared to commercial separators (Figure 11B). These separators demonstrate excellent cycling (93.2%) and rate capabilities (97.9%) in lithium-ion batteries, showing promise for future applications. Chen et al. [112] fabricated a hierarchical poly (ether sulfone) (PES) porous membrane for vanadium flow battery applications using a hard template method and achieved a controlled pore size for optimal conductivity and selectivity (Figure 11C). The optimized membrane demonstrated excellent electrochemical performance with a columbic efficiency of 94.52%, energy efficiency of 81.66%, and stability over extended cycling at 80 mA cm^−2^, highlighting its potential for use in VFBs due to its low cost and chemical stability.

In solid-state batteries, HPPMs serve as solid electrolytes or electrolyte scaffolds, providing pathways for ion conduction in both solid and quasi-solid environments. Wang et al. [113,114,115,116] have advanced the development of polymer based solid-state electrolytes (SSEs) for lithium and sodium metal batteries (LMBs and SMBs), with a focus on improving ionic conductivity and interfacial stability. The hierarchical porosity allows for enhanced ionic conductivity by facilitating the movement of ions through interconnected channels, even in the absence of a liquid electrolyte. The mechanical strength provided by the porous structure ensures that the membrane can withstand the stresses associated with volume changes during charging and discharging, which is a common issue in SSBs. These membranes address the challenges of low ionic conductivity and poor interfacial contact, which are often encountered in solid-state battery systems.

#### 3.1.2. Electrodes

**Figure 12:**
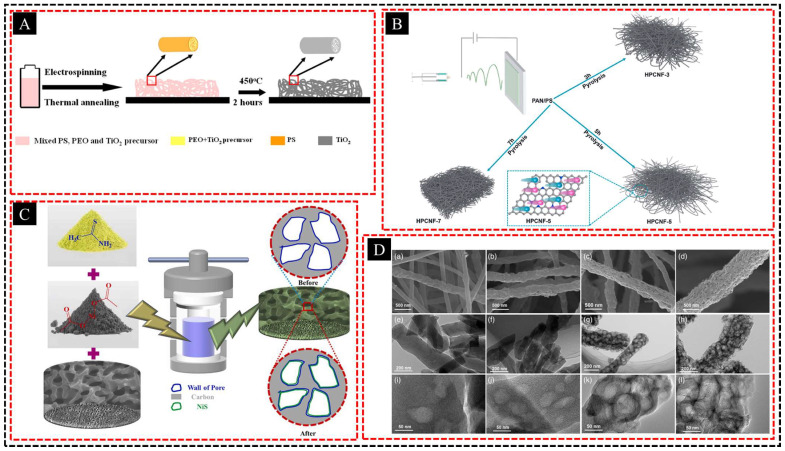
(**A**) Fabrication of TiO_2_ fibers prepared by electrospinning a blend solution of polystyrene-block-poly (ethylene oxide) (PS-b-PEO) and titanium-tetraisopropoxide. (**B**) Schematic illustration of the synthetic process via the electrospinning method. (**C**) Schematic illustration of the fabrication procedure of NiS@EM. (**D**) SEM image of (a) PCN-15, (b) PCN-30, (c) PCN-45, and (d) PCN-60. TEM images of (e) PCN-15, (f) PCN-30, (g) PCN-45, and (h) PCN-60. High-resolution TEM images of (i) PCN-15, (j) PCN-30, (k) PCN-45, and (l) PCN-60. Adapted with permission from Refs. [117,118,119,120]. Copyright 2013@American Chemical Society, Copyright 2022@Elsevier Publisher, Copyright 2023@Elsevier Publisher, Copyright 2021@ Wiley.

Hierarchically porous membranes are being increasingly explored for use in battery electrodes, particularly as precursors for the development of high-performance electrode materials. These membranes with their multiscale pore structures offer significant advantages when used as templates or scaffolds for electrode fabrication, especially PVP [101] and PAN [109]. By serving as precursors, porous membranes provide a framework that can be infused with active materials, such as metals, metal oxides, or carbon. Upon thermal treatment or chemical modification, membranes decompose or transform, leaving behind a well-structured porous electrode material that retains its hierarchical architecture. This unique structure promotes efficient ion and electron transport, enhances the active surface area, and improves the overall electrochemical performance, making it highly suitable for advanced battery applications, such as lithium-ion, lithium–sulfur, and solid-state batteries. You et al. [117] designed novel cigar-like nanofibers with an outer shell and inner continuous pore structure that were fabricated using a combination of PS-b-PEO self-assembly and electrospinning (Figure 12A). The block copolymer serves as a medium for the electrospinning process, a template for the formation of the cigar shape, and a potential agent for controlling the structure and size. Meanwhile, the TiO_2_ precursor helps regulate the fiber shape during the annealing and calcination processes. These high-surface-area fabrics exhibit excellent mechanical strength, superior charge/discharge capacity, and stability as Li-ion battery electrodes, owing to their hierarchical pore structures. Sun et al. [118] developed nitrogen-doped hierarchical porous carbon nanofibers (HPCNF-5) based on PAN/PS nanofibers via electrospinning and pyrolysis (Figure 12B), and the shape of those fibers can be well maintained through final pyrolysis, providing fast electron transfer and structural defects for enhanced capacitive behavior. As a flexible electrode for potassium-ion batteries, HPCNF-5 achieves high reversible capacity (282.9 mAh g^−1^ at 0.5 A g^−1^), excellent rate capability, and long cycle life, retaining 88% capacity after 2000 cycles. Liu et al. [119] successfully prepared a flexible electrode (NiS@EM) with nano-NiS uniformly distributed in a 3D porous membrane using hydrothermal synthesis (Figure 12C), thereby enhancing the sodium storage performance by exposing more reactive sites. As an anode in sodium-ion batteries, NiS@EM achieved a high reversible capacity of 658.3 mA h g^−1^ and demonstrated excel-lent rate and cycle stability, owing to its porous structure and improved utilization of nano-active materials. Su et al. [120] developed a flexible porous carbon nanofibrous membrane as an anode for sodium-ion batteries by electrospinning polyacrylonitrile (PAN) with ZIF-8 nanoparticles, followed by carbonization (Figure 12D). The resulting anode, with 45% ZIF-8, achieved a high Na storage capacity of 418 mA h g^−1^ and a plateau capacity of 278 mA h g^−1^, attributed to the adsorption-filling mechanism enabled by homogeneously dispersed micro–meso pores.

A major challenge in lithium–sulfur batteries is the polysulfide shuttle effect, where dissolved polysulfides migrate between electrodes, causing capacity fading and poor cycle stability. HPPMs can help address this issue by acting as functional separators that trap polysulfides within their pore structures, preventing their migration, and allowing for the smooth passage of lithium ions. These membranes can also host additional functional groups or coatings designed to interact with polysulfides, further mitigating the shuttle effect and improving the cycling performance of the LSBs. The hierarchical pores enhance ion transport and help control the polysulfide species, contributing to a longer battery life and better energy efficiency. Wu et al. [121] developed advanced hierarchical porous polymer nanosheets (AHPPNs) using a novel fabrication method that combined surface-initiated atom transfer radical polymerization with crosslinking-induced co-assembly. These nanosheets effectively suppressed poly-sulfide shuttling, immobilized sulfur, and enhanced ion/electron transport, resulting in significantly improved performance and cycling stability of lithium–sulfur batteries.

### 3.2. Supercapacitors

In supercapacitors, hierarchically porous membranes play a key role in enhancing the energy and power density by providing a high surface area for charge storage and facilitating fast ion diffusion [122,123,124]. The large surface area provided by the hierarchical pores increases the available surface area for ion adsorption, which is critical for EDLC-type supercapacitors. This increased the capacitance and energy storage capabilities. The interconnected pore network enables rapid electrolyte ion movement, reduces diffusion limitations, and improves rate capability. For supercapacitors with pseudocapacitive materials (e.g., transition metal oxides and conducting polymers), hierarchically porous membranes enhance the surface interaction between the electrolyte and active material. This allows more faradaic reactions to occur, thereby improving the energy density. In conductive membranes (e.g., carbon-based membranes), hierarchical pores enhance electron transport, facilitating better electrical conductivity.

**Figure 13:**
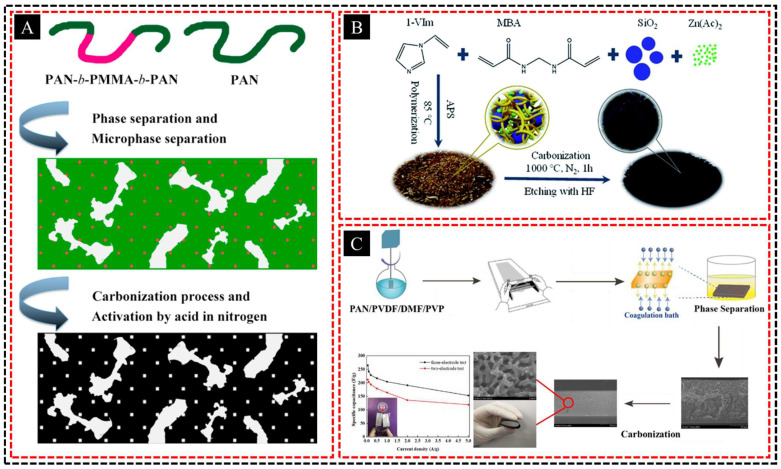
(**A**) Schematic illustration of the synthesis process of AHPC materials. (**B**) Schematic illustration of the synthesis of NSHPC. (**C**) Preparation of novel 3D hierarchical porous carbon membrane as flexible free-standing electrode for supercapacitors. Adapted with permission from Refs. [125,126,127]. Copyright 2016@Elsevier Publisher, Copyright 2019@Royal Society of Chemistry, Copyright 2020@Elsevier Publisher.

Ran et al. [125] synthesized the homopolymer PAN and triblock copolymer PAN-b-PMMA-b-PAN through RAFT polymerization, and used phase inversion, carbonization, and HNO_3_ activation to create activated hierarchical porous carbon membranes (Figure 13A). These membranes demonstrated excellent electrochemical performance with a specific capacitance of 297.0 F g^−1^, retaining 90% after 2000 cycles, and showed high energy (15.8 Wh Kg^−1^) and power density (4000 W Kg^−1^) in symmetric supercapacitors. Xu et al. [126] synthesized nitrogen and sulfur co-doped hierarchically porous carbon (NSHPC) via zinc acetate-assisted pyrolysis of polymer networks, achieving a high surface area of 1057 cm^2^ g^−1^ with both micro- and mesopores (Figure 13B). NSHPC shows excellent performance as a metal-free electrocatalyst for oxygen reduction, anode material for lithium-ion batteries with a stable capacity of up to 740 mA h g^−1^, and electrode material for supercapacitors with a capacitance of 203 F g^−1^. Liu et al. [127] developed a flexible 3D hierarchical porous carbon membrane for supercapacitor electrodes using PAN, PVDF, and PVP via nonsolvent-induced phase separation and carbonization (Figure 13C). These membranes demonstrated excellent flexibility, a large surface area (491 m^2^ g^−1^), and high specific capacitance of 265 F g^−1^ in a three-electrode system, attributed to their hierarchical porous structure and enhanced surface functional groups.

Supercapacitors benefit from the high surface area provided by HPPMs as it allows more effective charge storage at the electrode–electrolyte interface [128]. The multiscale pore structure ensures rapid ion transport, reduces internal resistance, and allows for faster charge-discharge cycles, which is a critical requirement for high-power-density applications. Moreover, the porous structure of these membranes enhances the overall energy density of supercapacitors by providing a larger area for electrochemical reactions to occur while maintaining mechanical stability during repeated cycling. This combination of high power and energy density makes them ideal for applications requiring rapid energy delivery and recovery.

In lithium-ion batteries, these membranes can serve as advanced separators [129] that offer high ionic conductivity and enhanced safety by preventing the growth of Li dendrites, which can lead to short circuits. In supercapacitors, the large surface area and efficient ion transport enabled by the porous structure lead to improved capacitance and energy density, thereby addressing one of the key challenges in these systems. Addition-ally, the tunability of the membrane’s pore size and chemical composition allows for customization according to specific application requirements, making it adaptable to emerging technologies such as solid-state batteries and lithium–sulfur batteries. Here, we summarize the commonly used polymer types and their combinations with hierarchical pore structures, along with their applications in various electrochemical energy storage devices, as shown in Table 1. In conclusion, HPPMs represent a promising solution for overcoming the performance limitations of current electrochemical energy storage devices. Their unique combination of high surface area, enhanced ion transport, improved electrolyte accessibility, and superior mechanical stability makes them attractive materials for various advanced energy storage applications. Researchers continue to explore and optimize these membranes as they are expected to play a pivotal role in the future of energy storage technologies, enabling devices with higher energy and power densities, greater efficiency, and longer operational lifetimes.

## 4. Recent Innovations of Hierarchically Porous Polymer Membranes

Several cutting-edge advancements in the development of HPPMs have reshaped the landscape, particularly in pore structure optimization, composite material fabrication, and sustainability [130,131]. These advancements are driven by the need for membranes that can deliver superior performance while addressing environmental concerns, making them essential for a range of industries, such as energy storage, filtration, and environmental protection.

### 4.1. Composite Membranes

**Figure 14:**
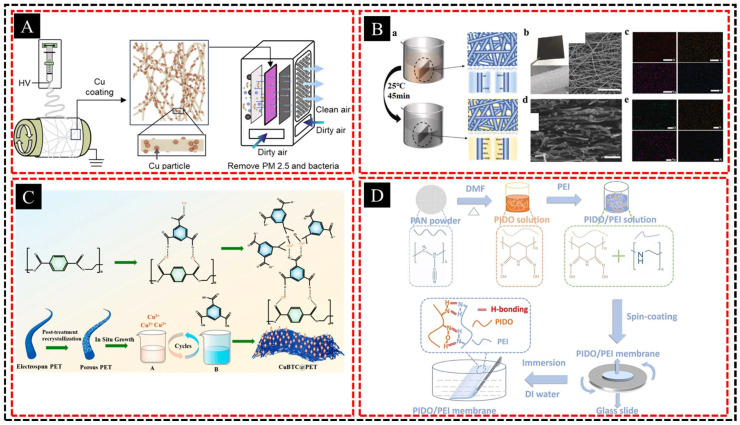
(**A**) Schematic Illustrating of hierarchically porous superhydrophobic PLLA/copper composite fibrous membranes for air filtration. (**B**) Synthesis and structural characterisation of the PDA/PSS@CNF/GO membrane. (a) Synthesis of the PDA/PSS@CNF/GO membrane. (b) PDA/PSS@CNF/GO membrane (upper left) and SEM of the PDA/PSS@CNF/GO membrane surface (right scale bar: 50 μm; left scale bar: 100 nm). (c) EDX results for the surface of the PDA/PSS@CNF/GO membrane (scale bar: 50 μm). (d) Cross-sectional SEM image of the PDA/PSS@CNF/GO membrane (scale bar: 10 μm). (e) EDX results for the cross-section of the PDA/PSS@CNF/GO membrane (scale bar: 10 μm). (**C**) Schematic route of CuBTC@PET preparation. (**D**) Schematic illustration for the fabrication process of PIDO/PEI membrane. Adapted with permission from Refs. [132,133,134,135]. Copyright 2024@American Chemical Society, Copyright 2024@Elsevier Publisher, Copyright 2024@MDPI, Copyright 2023@Elsevier Publisher.

Another area of significant progress is the development of composite membranes, in which phase separation is integrated with other fabrication techniques to produce materials with enhanced properties. Composite membranes combine the benefits of different materials, structures, and techniques to create stronger, more durable, and more functional membranes than their single-material counterparts. Huang et al. [132] developed a hierarchically porous PLLA membrane by electrospinning and acetone treatment with added copper particles for antimicrobial functionality (Figure 14A). The resulting PLLA/Cu composite membranes exhibit excellent air permeability, antimicrobial properties, and superhydrophobicity, making them ideal for high-flux filtration applications in healthcare and laboratory settings. Hao et al. [133] fabricated a composite membrane with enhanced ion transport by co-depositing polydopamine (PDA) and poly(sodium-p-styrenesulfonate) (PSSNa) onto a hierarchical 3D porous nanofiber substrate composed of carbon nanofibers and 3D graphene sheets (Figure 14B). By optimizing the PDA/PSS ratio, they achieved improved ion channel properties, leading to a power density of 7.5 W m^−2^ between seawater and river water. Tulugan et al. [134] developed functional hierarchically porous composite membranes by combining electro-spun PET with metal–organic frameworks (MOFs), specifically CuBTC, to enhance the performance of nanofiltration membranes (NFMs) (Figure 14C). The resulting superhydrophobic CuBTC@PET (SCP) fibers that were created using a low-cost in situ growth technique im-proved the stability of CuBTC in water, thus expanding its potential applications. Li et al. [135] developed an ultrathin, highly crosslinked PIDO/PEI composite membrane for the selective recovery of Pt from acidic solutions, offering abundant nitrogen-containing active sites and enhancing membrane uniformity (Figure 14D). The hierarchical porosity and acid tolerance of the membrane enabled efficient Pt (IV) extraction from the spent catalysts, and the Pt-loaded membrane could be converted into a fresh Pt/C catalyst with superior electrochemical performance.

Integrating phase separation with electrospinning allows the creation of membranes that feature both highly porous structures and nanofiber networks, thereby improving the mechanical strength while maintaining high permeability and selectivity. Additionally, chemical vapor deposition (CVD) can be used in conjunction with phase separation to coat membranes with functional layers such as hydrophobic or catalytic surfaces, which can further expand their range of applications [136,137]. Composite membranes are widely used in water treatment, gas separation, and biomedical applications, where a combination of tailored pore structures and surface functionalities enables superior performance in complex environments. This approach also opens doors to membranes with multifunctional capabilities, such as membranes that not only filter but also degrade contaminants or membranes with enhanced thermal stability for use in harsh conditions.

### 4.2. Environmentally Friendly Materials

**Figure 15:**
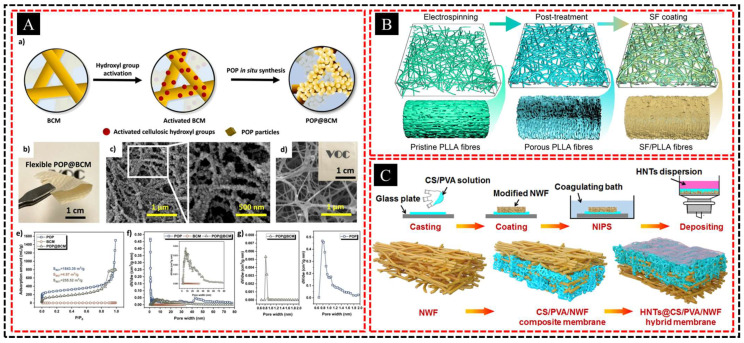
(**A**) Hierarchically porous bacterial cellulose nanofibrous membranes for selective adsorption and real-time colorimetric monitoring of volatile carboxylic acids. (a) Fabrication of hierarchical porous POP@BCM. (b) Optical images of flexible POP@BCM. SEM images of (c) POP@BCM, (d) BCM (the inserted figure shows the optical image of BCM). (e) N2 physisorption isotherms at 77 K and (f) pore size distributions of different materials (diameter ranges from 0.5–80 nm). (g) Pore size distribution at the microporous range (0.5–2 nm). (**B**) Schematics of the electrospun PLLA fibrous membrane, porous PLLA fibers after post-treatment and silk fibroin coating on PLLA fibers. (**C**) Schematic diagram for the fabrication process of the HNTs@CS/PVA/NWF hybrid membranes. Adapted with permission from Refs. [138,139,140]. Copyright 2024@ Springer Publisher, Copyright 2021@Elsevier Publisher, Copyright 2020@Elsevier Publisher.

As sustainability has become a critical priority across industries, the development of environmentally friendly materials for membrane fabrication is gaining traction. Traditional polymer membranes often rely on petrochemical-based materials and solvents that pose environmental challenges in terms of production and disposal. To address these concerns, researchers have focused on phase separation methods that utilize biodegradable or eco-friendly polymers to create membranes with a reduced environmental impact. These efforts include the use of renewable polymers such as polylactic acid (PLA) and cellulose derivatives, which degrade naturally and are sourced from sustainable materials. Tang et al. [138] designed a hierarchically porous bacterial cellulose nanofibrous membrane with an in situ-grown triazine-based porous organic polymer, achieving a structure with micro-, meso-, and macropores (Figure 15A). This composite demonstrated high flexibility, a large surface area (255 m^2^/g), and exceptional adsorption capacities for volatile carboxylic acids (VCAs), including formic acid (815.36 mg/g) and acetic acid (754.21 mg/g). Song et al. [139] developed a vascular scaffold by combining silk fibroin (SF) with highly porous electro-spun poly (l-lactic acid) (PLLA) membranes in which PLLA fibers were treated with acetone to create a porous structure (Figure 15B). The SF solution filled the pores and coated the PLLA fibers, resulting in hierarchical porous SF/PLLA membranes that demonstrated potential as effective scaffolds for muscle artery regeneration.

In addition, researchers are exploring the use of green solvents in phase separation processes to further minimize environmental harm. These biodegradable membranes are not only designed to reduce waste, but also to maintain high performance in applications such as water purification, where eco-friendly materials are critical for sustainable long-term use. Beyond their environmental benefits, biodegradable membranes can also offer new functionalities such as selective degradation in biological environments, making them suitable for medical applications such as drug delivery and tissue engineering. Wang et al. [140] developed an environmentally friendly hybrid membrane composed of halloysite nanotubes, chitosan, polyvinyl alcohol, and nonwoven fabric (HNTs@CS/PVA/NWF) for air filtration, utilizing a dip-coating method combined with nonsolvent-induced phase separation (Figure 15C). This reinforced composite membrane demonstrated excellent filtration performance owing to direct interception and strong interactions, achieving antibacterial rates of 99.6% against *E. coli* and 99.1% against *S. aureus*, which were attributed to the synergistic effects of the chitosan component and TiO_2_ nanoparticles.

In conclusion, the evolution of hierarchical porous membranes has been driven by innovations in pore structure design, composite material integration, and sustainable material development. These advancements have enabled the creation of more efficient, multifunctional, and eco-friendly membranes that can revolutionize industries ranging from water filtration and energy storage to healthcare and environmental remediation. By addressing both the performance and environmental concerns, these next-generation membranes represent a critical step toward meeting the growing global demand for high-performance and sustainable materials.

## 5. Challenges and Future Directions in Electrochemical Energy Storage

Looking ahead, the future of HPPMs in electrochemical energy storage devices is full of potential, driven by advancements in materials science and fabrication techniques. Research is increasingly focused on enhancing the performance, sustainability, and versatility of these membranes to meet the growing demands of energy storage technologies such as batteries, supercapacitors, and fuel cells [141,142]. A key trend in this area is the development of sustainable, biodegradable membranes as environmental concerns and regulations push for greener alternatives. The integration of renewable materials with innovative manufacturing processes is expected to result in membranes that not only improve device performance but also reduce environmental impact through lower waste and energy consumption.

In addition to sustainability, advances in nanotechnology and materials science are shaping the future of hierarchically porous membranes in energy storage. These advances are expected to lead to membranes with specialized functionalities, such as enhanced ionic conductivity, ion selectivity, or stimuli-responsive behavior, where the membrane can adapt its properties in response to changes in conditions like voltage or temperature [143,144]. Self-healing and self-cleaning membranes are also promising, potentially increasing the lifetime and reliability of energy storage devices by repairing structural defects or repelling contaminants that may impair performance over time.

However, several technical challenges must be addressed in the continued development of HPPMs for electrochemical energy storage applications:

### 5.1. Uniformity and Reproducibility

Achieving uniform pore size and structure over large membrane areas remains a critical challenge for electrochemical energy storage devices, where inconsistent pore formation can hinder ion transport, reduce energy density, and impair charge-discharge rates [145,146]. For applications such as battery separators or solid-state electrolyte membranes, pore uniformity is essential for maintaining consistent performance. Current research is focused on improving the reproducibility of phase separation processes and other fabrication techniques to ensure uniformity. Fine-tuning parameters like temperature, concentration, and solvent evaporation rates during membrane synthesis can help achieve more consistent results, thereby enhancing the overall performance of the energy storage system.

### 5.2. Scalability

Scaling up the production of hierarchically porous membranes for large-scale energy storage applications poses another significant challenge [147,148]. While lab-scale techniques often produce high-performance membranes with precisely controlled structures, translating these processes to industrial-scale production without sacrificing quality or functionality is difficult. In electrochemical energy storage, membrane scalability is crucial for commercial viability. Research is exploring how to adapt phase separation and other membrane fabrication techniques for mass production, including the use of continuous manufacturing methods and advanced automation technologies that maintain the structural integrity and electrochemical performance of the membranes at larger scales.

### 5.3. Advanced Functionalization

Incorporating advanced functionalities into HPPMs is an area of growing interest, particularly for improving the efficiency and versatility of energy storage devices [149,150]. Functionalizing these membranes during fabrication—such as by introducing conductive polymers, ion-conductive additives, or catalytic materials—can enhance ionic conductivity, electrochemical stability, and overall energy storage capacity. Functionalized membranes could find applications in next-generation batteries (e.g., lithium–sulfur or solid-state batteries), where improved ion transport and enhanced electrode–electrolyte interfaces are critical for achieving higher energy densities. Researchers are refining fabrication techniques to enable the seamless integration of these functional materials without compromising the membrane’s pore structure or mechanical stability.

Overall, the future of HPPMs in electrochemical energy storage devices will be shaped by progress in sustainability, scalability, and functionality. As research addresses these challenges, the field can expect the emergence of more efficient, eco-friendly, and commercially scalable membranes. These advancements will play a crucial role in the development of high-performance energy storage systems capable of supporting the growing demand for sustainable energy technologies in sectors such as electric vehicles, grid storage, and portable electronics [151,152].

## 6. Conclusions

Recent advancements in polymer hierarchically porous membranes have significantly enhanced their application potential in filtration, separation, and energy storage. These membranes, characterized by their hierarchically porous structures, optimize fluid dynamics and surface area, enabling efficient contaminant removal and improved energy storage capacity. Innovative fabrication techniques such as electrospinning and phase separation have allowed for precise customization of pore sizes, enhancing performance across various applications. As highlighted in this review, ongoing research will be crucial for unlocking the full potential of these materials, leading to more sustainable and efficient technologies in the future.

## Figures and Tables

**Table 1:** Common polymer types with hierarchical pore structures and their applications in electrochemical energy storage devices.

Applications	Types	Pore Structures	Part
Batteries	PVP [101]	Micropores + mesopores + macropores	Electrode
	SPEEK [102]	Macropores + mesopores	Electrolyte
	PAN [104]	Macropores + mesopores	Separator
	TPY-CNF + PVP/PAN [105]	Macropores + nanopores	Separator
	Poly (ionic liquid) [106]	Macropores + mesopores	Electrolyte
	Aramid nanofiber [107]	Microporous + nanopores	Separator
	PVDF [108]	Macropores + mesopores	Separator
	PAN [109]	Macropores + mesopores	Electrode
	PA6/PET/PA6 [110]	Macropores + macroscale pores	Separator
	PVDF/PMIA [111]	Macropores + mesopores	Separator
	PES [112]	Finger-like pores + nanoscale pores	Separator
	PANI/PI [129]	Micropores + nanopores	Separators
Supercapacitors	PANI [98]	Microchannels + Macropores	Electrode
	GDY/PVA [123]	micropore	Separator
	PAN-b-PMMA-b-PAN triblock copolymer [125]	Micropores + mesopores	Electrode
	Poly(1-vinylimidazole) [126]	Micropores + mesopores	Electrode
	PAN, PVDF, PVP [127]	Micropores + mesopores	Electrode
	PS-b-P2VP block copolymer [128]	Micropores + mesopores	Electrolyte

## Data Availability

The data that support the findings of this study are available on request from the corresponding author upon reasonable request.
